# Multi-Analyte Network Markers for Tumor Prognosis

**DOI:** 10.1371/journal.pone.0052973

**Published:** 2012-12-26

**Authors:** Jongkwang Kim, Long Gao, Kai Tan

**Affiliations:** 1 Department of Internal Medicine, University of Iowa, Iowa City, Iowa, United States of America; 2 Department of Biomedical Engineering, University of Iowa, Iowa City, Iowa, United States of America; Vanderbilt University Medical Center, United States of America

## Abstract

Deregulation of gene expression, a hallmark of cancer, is caused by both genetic and epigenetic mechanisms. The rapid accumulation of epigenome maps of various cancers suggests a new avenue of research, namely integrating epigenomic data with other types of omic data for cancer diagnosis, prognosis, and biomarker discovery. We introduce the MAPIT algorithm (Multi Analyte Pathway Inference Tool), to enable principled integration of epigenomic, transcriptomic, and protein interactome data. As a proof-of-principle, we apply MAPIT to glioblastoma multiforme (GBM), the most common and aggressive form of brain tumor. Few predictive markers were reported for the prognosis of GBM patients. By integrating mRNA transcriptome, promoter DNA methylome and protein-protein physical interactome, we find ten expression- and three methylation-based network markers, involving 118 genes. When tested on additional GBM patient samples, the prognostic accuracy of the multi-analyte network markers (73.5%) is 9.7% and 8.6% higher than previous prognostic signatures built on gene expression or DNA methylation alone. Our results highlight the critical role of two novel pathways in the prognosis of GBM patients, small GTPase-mediated protein trafficking and ubiquitination-dependent protein degradation. A better understanding of these two pathways could lead to personalized therapies for subgroups of GBM patients. Our study demonstrates that integrating epigenomic, transcriptomic, and interactomic data can improve the accuracy network-based prognosis markers and lead to novel mechanistic understanding of cancer.

## Introduction

Glioblastoma multiforme (GBM) is the highest grade tumor of astrocytes (WHO grade IV [1]). It is also the most common and lethal form of brain tumor with a median survival time of 12–15 months after initial diagnosis [2], [3]. In spite of a short median survival time, a small percentage of GBM patients can live a very long time (3–20 years) after diagnosis. In this study, we call these patients Long Term Survivors of GBM (LTS-GBM). Understanding the molecular pathways that distinguish these rare LTS patients from Short Term Survivors (STS) could lead to more effective treatment and management of the deadly disease.

Few predictive gene markers for GBM patient outcome were reported until recently. Using four independently collected sets of gene expression profiles, Colman *et al.* found a set of 38 genes that can distinguish STS (median survival time = 39 weeks) from LTS patients (median survival time = 146 weeks) [4]. Using another compendium of gene expression profiles generated by The Cancer Genome Atlas (TCGA) consortium, Verhaak *et al.* [5] classified GBM patients into four subtypes based on their gene expression profiles. They found a trend towards longer survival among patients with a proneural subtype although the trend is not statistically significant. More recently, using a compendium of CpG island DNA methylation profiles generated by the TCGA consortium, Noushmehr *et al.* identified a CpG island methylator phenotype (involving 1,228 gene promoters) that are associated with significantly improved disease outcome [6].

Diseases of complex etiology such as cancer are consequences of combined defects of many genes. These disease genes in turn drive the pathogenesis through an integrated network response. Thus, the historical approach of investigating disease by studying individual genes and linear pathways must be complemented by a systems biology approach that will more likely identify nodal points affecting network dynamics, yielding targets of strong therapeutic potential. The large-scale generation and integration of genomic, transcriptomic, proteomic, and metabolomic data have enabled the construction of complex gene networks that provide a new framework for understanding the molecular mechanism of diseases. This network-based view of disease is profoundly different from the familiar linear causality model that generally fails to account for the complexity of human biology and the intricate web of interactions associated with a particular disease phenotype.

A number of studies have shown that network-based markers provide a more effective and accurate means for cancer gene discovery and disease subtype stratification. Additionally, compared to traditional approaches that do not explicitly consider relationships between genes/proteins in a pathway, the network-based approach naturally provides a mechanistic understanding of the underlying pathways. Chuang *et al.* [7], Taylor *et al.* [8], and Lefebvre *et al.* [9] integrated gene expression profiles with physical protein-protein interactome data to identify subnetwork markers for the prognosis of breast cancer and lymphoma patients. Torkamani and Schork [10] used gene co-expression network to infer cancer-initiating genes in breast, colorectal cancer, and glioblastoma. Although highly promising, none of these previous studies incorporated epigenetic data into their integrative analyses in spite of the well-established critical role of epigenetics in cancer etiology [11], [12]. For the sake of discussion, we termed those previous approaches using gene expression data only as single-analyte network based approach.

Both histone tail post-translational modification and DNA methylation have been shown to play a critical role in tumorigenesis and progression [13], [14]. For instance, hypermethylation of the genes encoding NSD1, the death-associated protein kinase DAPK, epithelial membrane protein-3, and CDKN2A has been linked to poor outcomes in neuroblastoma, lung, brain and colorectal cancers, respectively [13]. For GBM, promoter hypermethylation of the *MGMT* gene (O6-methylguanine methyltransferase) has been linked to poor disease outcome [15], [16]. While promising, it is likely additional methylation-based biomarkers could complement *MGMT* status as an outcome predictor. The advent of next-generation sequencing and high throughput tandem mass spectrometry has enabled epigenomic profiles to be generated at unprecedented rate for various types of cancers. Clustering analysis of epigenomic data has revealed prognostic signatures that are complementary to gene expression patterns [6]. Recently, Wen et al. [17] has reported an integrative analysis of transcriptomic, epigenomic, and protein interactome data to discover driver genes in colorectal cancer. They used DNA methylation data as prior information for candidate driver genes. However, a similar integrative analysis has not been conducted to identify prognostic markers for cancers.

We hypothesize that multi-analyte network markers can be discovered by integrating gene expression profile, epigenomic profile, and protein-protein interactome. These markers can be used to improve cancer prognosis accuracy compared to previous approaches in which only transcriptome and interactome data are integrated. To this end, we develop a novel computational framework that enables principled integration of multi-dimensional genomic and interactome data for molecular pathway inference. We implement the framework in the MAPIT (Multi Analyte Pathway Inference Tool) algorithm. We apply the MAPIT algorithm to identify prognostic network markers to predict GBM patient survival time. Our integrated analysis reveals that genes involved in protein trafficking, apoptosis, and protein catabolism play a critical role in predicting GBM patient outcome.

## Materials and Methods

### Classification of GBM patients based on their survival time

There is no a clear-cut and universal definition of LTS-GBM. In this study, we used the definition by Colman *et al.* [4], i.e. a patient is classified as a LTS if s/he survives at least two years after the initial diagnosis. Using this criterion, we have identified a total of 42 LTS and 237 STS patients from the TCGA data set. Patient clinical information is provided in Figure S1 and Table S1.

### GBM Patient Gene Expression and CpG island DNA Methylation Data

We downloaded gene expression and promoter DNA methylation data for 279 GBM patient samples from the TCGA data portal. Matching clinical data such as survival time after diagnosis were also obtained from TCGA. Gene expression profiling was done using the Agilent G4502A platform covering 17,814 genes. Promoter CpG island methylation profiling was done using the Illumina Infinium HumanMethylation27 platform, covering 13,372 genes. There are additional methylation data generated by the TCGA using two other Illumina platforms. We only used data generated by the 27k platform because only this set has triplicate data for every patient sample. The other two sets are smaller and only have duplicate data. Some genes can have multiple promoters and a representative promoter with the most significantly differential methylation between LTS and STS patients was used. The number of genes shared by the two platforms was 12,872.

### Human Protein-Protein Interaction Data

We obtained experimentally derived, non-redundant protein-protein interaction data from the iRefIndex database (version 4.0) [18], which consolidates a number of primary protein interaction databases including BIND, BioGRID, CORUM, DIP, HPRD, IntAct, MINT, MPact, MPPI and OPHID. We also included the human MAP kinase interactome recently mapped by Bandyopadhyay *et al.* [19]. The final combined network contains 10,691 proteins and 47,162 interactions. A Venn diagram for the expression, methylation, and PPI datasets are shown in Figure S2.

### Selection of training set for prognostic network module discovery

In order to obtain the most characteristic samples from LTS and STS patients, we selected extreme samples from each subgroup to form the training set. Such an approach has been shown to improve the prognostic accuracy of gene signatures for several cancers [20]–[22]. The resulting set contains the top 21 longest surviving individuals (greater than 2.5 years survival time) and the bottom 21 shortest surviving individuals (less than 0.5 years). We used the same set of patients for both expression- and methylation-based network module discoveries.

### Construction of input networks

Using unique HUGO Gene Nomenclature Committee (HGNC) gene IDs, we mapped gene IDs from expression, DNA methylation, and PPI data and found 8,461 genes that are common among three datasets. The giant connected component of PPI network involves 8,171 proteins and 47,162 interactions (Figure S2), which used for all analyses described in this paper.

Next, we combined either expression or methylation profiles with the PPI network to construct two edge-weighted networks. First, for each gene *i* in the network, a q-value of differential expression/methylation between LTS and STS samples was computed using the SAM method [23]. The following equation is used to assign edge weight:
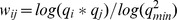
Where 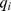 and 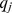 are SAM q-values for gene *i* and *j*, respectively and 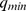 is the smallest q-value among all 8,171 genes in the network.

### Network module search using the miPALM algorithm

We recently developed a network module finding algorithm, miPALM, using un-weighted PPI networks [24]. The algorithm introduced a novel parameterised local modularity measure as its scoring function. Here, we extended miPALM to handle weighted networks. The algorithm starts by generating a ranked list of triangle seeds based on average edge weights. Starting from the top-ranked seed, *S* = {s,*t,u*}, the algorithm uses a greedy search strategy to expand it to a larger sub-network *S′* = {*s,t,u,v*}. The greedy search always merges the nearest neighbour *v* of *S* that leads to the largest increase in the local modularity measure, defined as
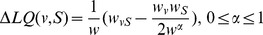
where 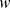 is the total edge weight of all nodes in the network, 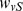 is the sum of edge weights between node *v* and all nodes in *S*, 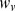 is the sum of edge weights attached to node *v*, 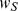 is the sum of edge weights attached to any node in *S*, and α is the parameter controlling the size of the neighbourhood of *S*. The seed expansion step repeats until no additional neighbour exists that can lead to an increase in the local modularity. Once a candidate subnetwork *S* is found, its final score is calculated as 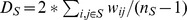, where 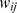 is edge weight and 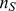 is the number of genes in subnetwork *S*. Note the score is normalized by the size of the subnetwork.

### Merging of overlapped subnetworks

Overlap score between two subnetworks was defined as 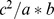, where *a* and *b* are the number of genes in the two subnetworks and *c* is the number of shared genes. We merged pairs of subnetworks if their overlap score is greater than 0.5.

### Significance assessment of network modules selected as classifier features

We generated 100 sets of random networks by permuting node weights of the the input network while maintaining the degree of each node. This permutation uncorrelates expression/methylation level with protein interactions. The miPALM algorithm was then run on the random networks. An empirical p-value of a candidate subnetwork was computed as the fraction of subnetworks found in the random networks with a score at least as large as that of the candidate subnetwork. A p-value cutoff of 0.05 was used to select significant subnetworks.

### GBM patient classification using Support Vector Machine (SVM)

Following the approach by Chuang *et al.* [7], we first normalized the expression or methylation level of a gene *i* across patient samples to obtain a gene-wise z-score, 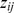. Given a patient sample *j*, the activity score of a network module *s* was calculated as 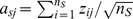 , where 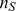 is the number of genes in the module. A feature vector was constructed as 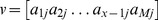 where *M* is the number of modules. Next, a SVM classifier with a quadratic kernel function was trained on the feature vectors derived from the LTS- and STS-GBM patient data in the training set.

### Classification accuracy assessment by leave-one-out cross validation (LOOCV)

Classification accuracy of the trained SVM classifier was tested on a test set of GBM patients not used in deriving the modules and training the classifier. Since there were only 21 LTS and 216 STS patients not used in the training step, our choice of LTS data for cross validation was limited. As a result, we generated a testing set by selecting 21 different STS patients and combining them with the same 21 LTS patients. Next, at each LOOCV iteration, data of 20 LTS and 20 STS patients were used to train the classifier and data of the remaining two patients were used for testing. The classification accuracy was defined as the ratio of the number of correctly classified patients to the total number of patients in the test set. We repeated the entire LOOCV procedure 100 times by using 100 test sets, each of which consisted of 21 randomly selected STS patients and the same 21 LTS patients from the set of GBM patients not used in training the classifier. The final classification accuracy reported is the average accuracy of the LOOCV runs.

### Feature selection by Recursive Feature Elimination

To identify a subset of highly discriminative modules, we devised a recursive module selection procedure based on the Recursive Feature Elimination (RFE) algorithm proposed by [25]. Briefly, the algorithm starts with the full set of significant modules and each module is regarded as a feature. At each iteration, a SVM classifier was trained using currently available features and the classification accuracy was estimated using cross validation. At the end of each iteration, each feature is assigned a weight by the SVM. The weight is a measure of the feature's contribution to the classification performance and can be used to rank them. The feature with the smallest ranking was removed at the end of each iteration. The algorithm terminates when there is no feature left in the training set. The subset of modules that gives the highest classification accuracy was selected as the final set. We examined a range of the alpha parameter values of the miPALM algorithm to identify the optimal alpha value that when combined with SVM classifier gave the largest classification accuracy. Figure S3 shows the results of parameter selection process.

### Other gene sets used in the study

The 38-gene set was obtained from [4]. The G-CIMP+ gene set was obtained from [6]. The COSMIC database [26] is a manually curated database containing human genes with somatic mutations that are observed in tumor samples and reported in scientific literature. From COSMIC, We obtained a list of 1,175 mutated genes observed in grade IV astrocytoma (GBM) samples.

## Results and Discussion

### Gene-expression-based network markers improve prognosis accuracy compared to markers without network information

GBM is the most aggressive form of tumor with less than 15% patients surviving more than 2 years after initial prognosis. Using a commonly used cutoff of two years [4], we have identified a total of 42 LTS and 237 STS patients from the TCGA data set. To characterize the effectiveness of the single-analyte (i.e. gene expression only) network approach on GBM patient prognosis, we integrated the gene expression data generated by the TCGA consortium with a set of non-redundant, experimentally derived human protein-protein interactions to construct a gene expression-informed network. For simplicity, we termed this network the *eNetwork*. Node weights in the eNetwork indicate the significance of differential gene expression between LTS- and STS- GBM samples. Under this scoring scheme, a deregulated pathway will manifest itself as a set of connected nodes (i.e. subnetworks) that collectively have a significantly large sum of node weights. To search for such high-scoring subnetworks, we extended our recently developed miPLAM algorithm for gene module finding [24] to handle weighted networks. Using the extended algorithm and a p-value cutoff of 0.05 (see Methods for p-value calculation of network markers), we found 65 network markers that are differentially expressed between LTS- and STS- GBM patients. For brevity, we termed these expression-based subnetwork markers *eModules*. Next, we used this set of eModules to train a statistical classifier for discriminating between LTS- and STS- GBM patients. Each GBM patient in the training set was represented by a profile of 65 eModule activity scores, one score from each eModule. Network activity profiles of 42 GBM patients (see Methods for the preparation of the training set) were used to train a Support Vector Machine (SVM) classifier. Since not all eModules are equally discriminative, we used an iterative feature selection procedure during SVM training to identify a subset of eModules that is most discriminative between LTS and STS patients (see Methods for details). By doing so, we identified a subset of 25 eModules (156 genes) that were most discriminative (i.e. achieving highest classification accuracy) (Table S2). Our final classifier was built using these 25 eModules. In the rest of this section, we will focus on these 25 eModules.

**Figure 1:**
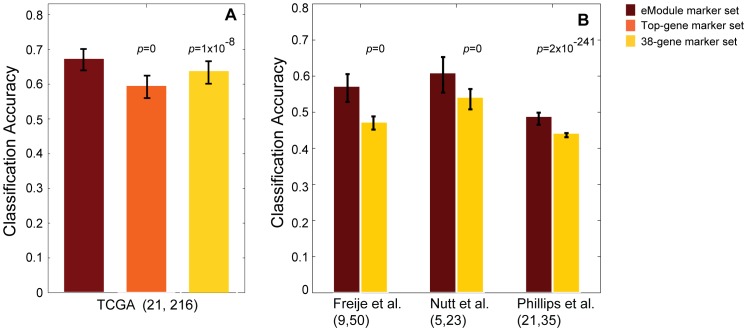
Performance comparison of gene-expression-based classifiers for GBM patient prognosis. **A**) Prognostic accuracy of various marker sets. Classification accuracy is defined as the ratio of the number of correctly classified patients to the total number of patients tested. Expression data of 42 GBM patients was used to derive the eModule set. Top-gene set is top 156 (size-matched to the number of genes in the eModule set) most significantly differentially expressed genes between LTS and STS patients. 38-gene set, a set of 38 discriminative genes reported in [4]. Two hundred thirty seven additional GBM patients from TCGA were used for testing classification accuracy. Error bar is the standard deviation based on 100 leave-one-out cross validations. **B**) Performance of eModule set and 38-gene set using three external microarray data [28]–[30] from which the 38-gene signature was derived. Numbers in parenthesis indicate number of LTS and STS in each data set, respectively. P-values are based on t-tests comparing the average classification accuracy of the eModule-based classifier and those of other classifiers.

Next, we tested the classification accuracy of the trained classifier using leave-one-out cross validation (LOOCV) and GBM patient samples that were not used in the derivation of the eModules (see Materials and Methods for details). We compared the average classification accuracy by eModule-based predictor to predictors built using two alternative sets of prognostic markers: the 38-gene set recently reported by Colman *et al* [4] and the set of top 156 (the same number of genes in the eModule set) most significantly differentially expressed genes between LTS and STS patients. As shown in Figure 1A, the average prognosis accuracy of the eModule-based classifier was 3.8% and 7.8% higher than the two alternative classifiers, respectively (t-test p<0.01).

While cross validation is a commonly used internal validation strategy, we further tested the performance of the eModule-based classifier using a more stringent approach, i.e. use of external data sets [27]. To this end, we used three additional independent gene expression data sets from which the 38-gene signature was derived [28]–[30]. The number of GBM patients ranges from 28 to 59 across the three data sets. For the classification, we used exactly the same classifiers trained on either the TCGA data (this study) or by other studies. As shown in Figure 1B, the eModule-based classifier significantly outperformed the 38-gene signature in all three external data sets (t-test p<0.01), suggesting that the improved classification accuracy of the eModule-based classifier is not due to biases in our experimental design.

In summary, by using both cross validation and external data sets, we found that eModule-based classifier provides improved prognosis accuracy of GBM patients compared to classifiers built without explicit consideration of relationships among genes.

### Low overall correlation between transcriptome and DNA methylome profiles in GBM samples

Accumulating evidence suggest that the relationship between promoter DNA methylation and gene expression is far more complicated than the classical view of anti-correlation between the two processes [31]. To better understand the relationship between gene expression and DNA methylation in the context of GBM, we conducted a global correlation analysis between the two types of data across the full set of GBM samples (N = 279). The median Pearson correlations between the expression and methylation profiles of 2,009 differentially expressed and 1,877 differentially methylated genes between LTS and STS groups (SAM test q<0.05) were −0.05 and −0.06, respectively. The average correlation between expression and methylation for the 156 genes in the set of eModules was −0.09. In comparison, the average correlation of a set of 1,877 randomly selected genes was −0.04 (Figure S4). Although on average the differentially changed genes showed higher negative correlation than random genes, the difference is rather moderate. This overall low correlation is unlikely due to poor data quality since both gene expression and DNA methylation data were generated using the same biological samples. Recently, Fan *et al.* conducted a meta-analysis of CpG island methylation data of twelve human tissues generated by the Human Epigenome Project using bisulfite sequencing [32]. They also observed a low correlation between promoter DNA methylation level and gene expression across tissues. Recently, CpG shore (sequence up to 2K bp distant from CpG island) rather than CpG island methylation has been shown to be more negatively correlated with gene expression in human cancers [33]. Unfortunately, the Illumina 27k platform used by the GBM DNA methylation study does not include probes for CpG shores. Future investigation using higher coverage data may provide additional insight into the low correlation between promoter DNA methylation and gene expression.

### DNA methylation-based network markers provide complementary pathway information for GBM patient prognosis

The overall low correlation between gene expression and DNA methylation profiles prompted us to examine if subnetwork markers based on DNA methylation profiles alone can provide complementary information for dissecting deregulated pathways in GBM. It has been well established that members of cancer pathways tend to have correlated expression and possess characteristic topological properties in the protein network, such as higher number of interacting partners and tendency of being centrally located in the network [34], [35]. A recent study shows that cancer-related genes tend to have correlated methylation profiles [36]. Further, in our own data, we observed that genes encoding connected protein pairs in the PPI network have significantly higher methylation profile correlation than genes encoding random pairs of proteins in the network (p = 2.2×10^−16^, Figure S5). Together, these observations provide additional rationale that methylation-based network markers could also be used for cancer prognosis.

Using the same strategy as with gene expression data, we constructed an alternative network by combining the PPI interactome with promoter DNA methylation profiles. We termed this network the *mNetwork* for brevity. Node values in the mNetwork indicate the significance of differential promoter methylation between LTS- and STS- GBM samples. We then applied the miPALM algorithm combined with the RFE feature selection strategy described above to identify discriminative subnetworks that are significantly differentially methylated between LTS and STS GBM patients. Using a p-value cutoff of 0.05, we found 7 such subnetworks involving 38 genes (Table S3). To contrast with the expression-based eModules, we termed these subnetworks *mModules*.

**Figure 2:**
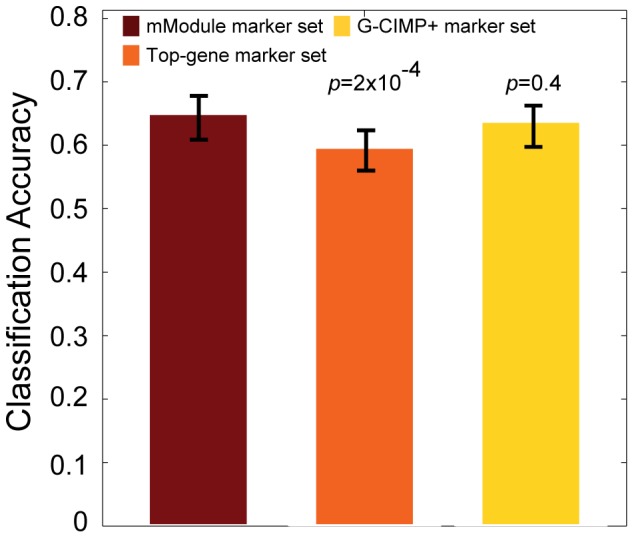
Performance comparison of DNA methylation-based classifiers for GBM patient prognosis. Promoter DNA methylation data of 42 GBM patients was used to derive the set of mModules. Top-gene set is top 38 (size-matched to the mModule set) most significantly differentially methylated genes between LTS and STS GBM patients. G-CIMP+ set, a set of 1228 discriminative genes reported in [6]. Two hundred thirty seven additional GBM patients from TCGA were used for testing classification accuracy. Error bar is the standard deviation based on 100 leave-one-out cross validations. P-values are based on t-tests comparing the average classification accuraciy of the mModule-based classifier and those of other classifiers.

Next, using leave-one-out cross validation, we compared the performance of our mModule-based predictor to predictors built with two alternative sets of prognostic markers: the G-CIMP+ predictor (1,228 gene promoters) recently reported by Noushmehr *et al.* [6] and the set of top 38 gene promoters (the same number of genes in the mModule set) that were most significantly differentially methylated between LTS and STS patients. As shown in Figure 2, we found that the mModule-based classifier slightly out-performed both the G-CIMP+ based predictor and the top-gene-based predictor. The average prognosis accuracies of the three classifiers based on LOOCV were 0.65, 0.64, and 0.62, respectively. The performance difference between the mModule-based and the G-CIMP+ based predictors was small, likely due to the fact that a much larger number of genes was used in building the G-CIMP+ based classifier than our mModule-based classifier (1,228 vs. 38),

We were unable to evaluate the prognosis accuracy of the mModule-based classifier using external datasets since additional sets of matched DNA methylation and patient survival time data were not available yet. However, since the module search algorithm is the same for eModule and mModule, it is reasonable to speculate that our mModule set will have similar prognostic value for additional DNA methylation data in the future.

Because of the connection between promoter DNA methylation and gene expression, we expected to find a reasonable overlap between the two sets of subnetwork markers. Surprisingly, we found a very low degree of overlap between genes in the two marker sets. Of the 156 eModule and 38 mModule genes, only five genes are shared between the two sets. This small overlap is unlikely due to poor quality of the identified modules because both sets of modules are supported by additional lines of evidence. For instance, the two sets of modules together captured 33 genes with reported somatic mutations in GBM patients [26]. But none of those genes are captured by both eModule and mModule sets. In summary, our data suggest that eModules and mModules are complementary to each other and represent different molecular pathways in gene network that is deregulated in GBM patients.

### Combining expression and methylation network markers results in large improvement of prognosis accuracy of GBM patients

**Figure 3:**
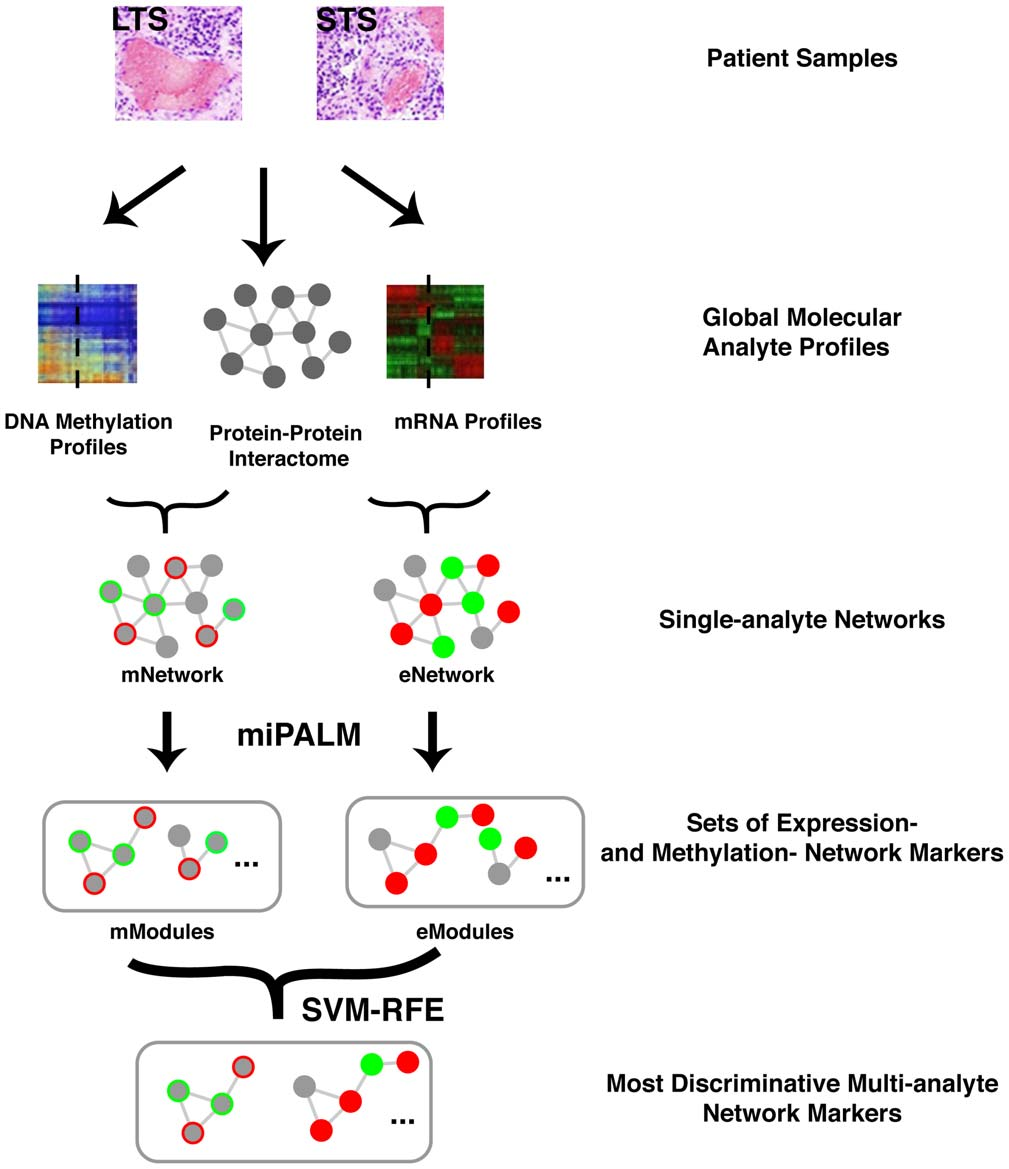
Overview of the MAPIT algorithm. Using clinical data, GBM patients are classified as either Long Term Survivors (LTS, >2 yrs.) or Short Term Survivors (STS, <2 yrs.). Two types of global measures of tumor samples are combined with protein-protein interactome (PPI) for network module identification: mRNA expression and promoter DNA methylation. Significance of change in either gene expression or promoter DNA methylation profiles between LTS and STS patients are overlaid on top of the PPI network to generate single-analyte networks, eNetwork and mNetwork. Network modules from each single-analyte network are identified using the extended miPALM algorithm [24] independently. Significant modules from each network are then combined to train a classifier for GBM prognosis using Support Vector Machine (SVM). A Recursive Feature Elimination (RFE) algorithm is implemented with the SVM classifier to select a final set of most discriminative network modules for patient prognosis.

Given the low degree of overlap between otherwise high-quality eModule and mModule sets and the moderate performance gain of mModule-based classifier alone, we asked if combining the two sets of heterogeneous pathway markers could lead to a more accurate predictor for GBM patient outcome compared to using only one type of pathway markers. Towards this goal, we developed the MAPIT algorithm (Multi-Analyte Pathway Inference Tool) for constructing a multi-analyte network marker-based classifier for GBM patient prognosis. Figure 3 provides an overview of the algorithm. Starting with the eNetwork and mNetwork, we first apply the miPALM algorithm to each input network separately to generate a set of eModules and a set of mModules. We then merge overlapping modules from the two sets. Next, for each merged module, two activity scores are calculated based on member gene expression and DNA methylation data, respectively. Both activity scores are then used as two independent features for building a statistical classifier using SVM combined with the RFE feature selection procedure. The MAPIT algorithm is implemented in Matlab and is freely available from our website http://www.healthcare.uiowa.edu/labs/tan/MAPITWebpage.html.

**Figure 4:**
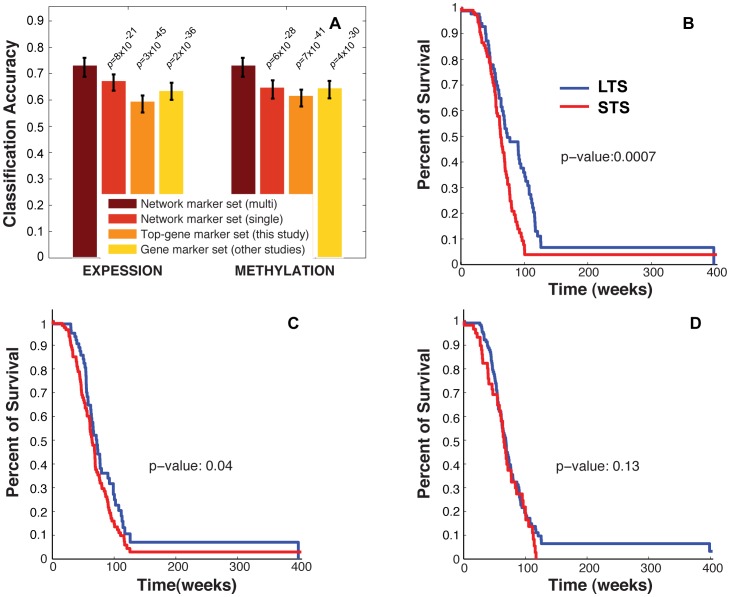
Performance of multi-analyte modules for GBM patient prognosis. **A**) Prognostic accuracies of GBM patients by four marker sets. Expression data of 42 GBM patient was used to derive the module set. Two hundred thirty seven additional GBM patients from TCGA were used for classification using the module set. Support Vector Machine algorithm was used to build a classifier based on each marker set. Top-gene sets were size-matched to the network module sets (i.e., the same number of genes as in the network module sets). Error bar is the standard deviation based on 100 leave-one-out cross validations. P-values are based on t-tests comparing the average classification accuracy of the multi-analyte-module-based classifier to those of other classifiers. **B–D**) Kaplan-Meier survival curves for LTS and STS GBM patients classified using the combined module set (B), 38-gene set (C), and G-CIMP+ set (D). P-value indicates the significance of separation between the two curves and is computed using logrank test.

We identified ten eModules and three mModules (118 total genes) that are highly discriminative of GBM patient subgroups. The classification accuracy based on leave-one-out cross validation (73.5%) using the combined subnetwork markers significantly improved over both single-analyte subnetwork markers and gene-set-based markers (Figure 4A). Additionally, the Kaplan-Meier survival curve showed more significant separation between the two patient groups classified by the multi-analyte network module set compared to previously reported 38-gene and G-CIMP+ signatures (Figure 4B–D).

Combining classification accuracy and significance of separation of patient survival time curves (Figure 4), we can draw two conclusions. First, the classifier built on multi-analyte network modules performed better than classifiers that are based on single-analyte network modules. Second, the network module-based classifier performed better than classifiers built on gene sets identified in previous studies.

We further corroborated our set of combined modules with four sets of genes implicated in GBM tumorigenesis: genes having somatic mutations in GBM patients from the COSMIC database [26], genes proposed to be prognostic markers for GBM patient survival time in two previous studies [4], [6], and genes in Copy Number Variation (CNV) regions identified in GBM patients [37]. We note that the presence of genetic mutations does not necessarily mean a gene is prognostic of cancer subtypes unless the mutations occur exclusively in one subtype. This kind of information is not yet available for most genes across large patient cohorts. However, it increases the likelihood of a module gene being a prognostic marker. We found that ten out of thirteen modules contain at least one gene that overlaps the published sets of GBM-related genes and the fraction of overlap with previous evidence ranges from 0.1 to 0.5 among the modules (Table S4). In total, 21 module genes (17.8%) overlap with published gene sets.

**Figure 5:**
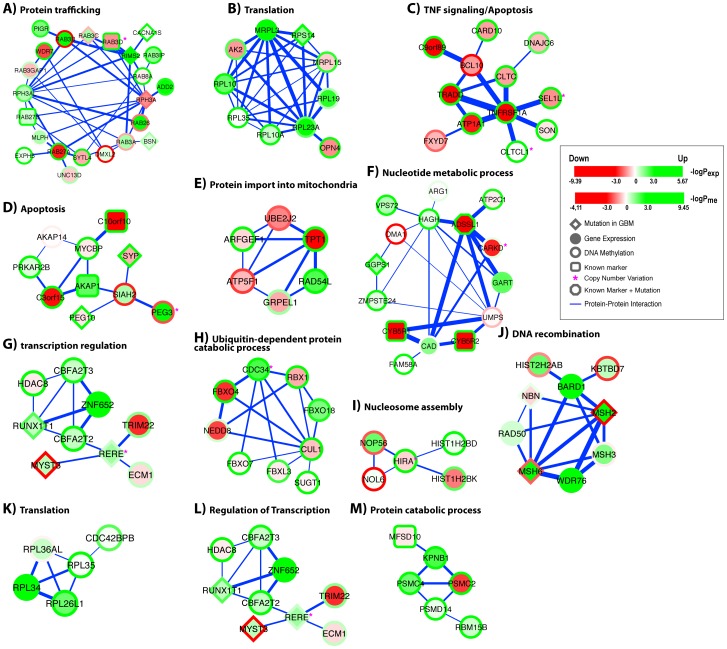
The set of multi-analyte prognostic modules identified by the MAPIT algorithm. Ten eModules (*A–J*) and three mModules (*K–M*) are shown. Node colour represents gene expression change of LTS patients compared to STS patients. Red, down-regulation; Green, up-regulation. Shade is proportional to the −log (p-value) of the change. Node border colour represents DNA methylation change of LTS patients compared to STS patients. Red, hypomethylation; Green, hypermethylation. Shade is proportional to the −log (p-value) of the change. Diamond nodes, genes reported to bear somatic mutations in GBM patients. Rectangular nodes, genes identified as GBM prognostic markers in either [4] or [6]. Hexagonal nodes, genes both reported to bear somatic mutations and identified as prognostic markers in either [4] or [6]. Purple star: genes located in CNV regions identified in GBM patients [37]. Edge, protein-protein interaction. Edge width is proportional to the combined significance of expression changes of the two involved nodes (see Methods for details).

The set of thirteen modules with their enriched GO terms (p<0.05) were depicted in Figure 5. Each node in a module displays expression and promoter DNA methylation information simultaneously for the gene represented by the node. For instance, *RAB3D* in module *A* shows down-regulated expression and hypermethylated promoter in LTS patients compared to STS patients. In decreasing number of module genes involved, the set of enriched GO terms consists of protein trafficking, apoptosis, protein catabolism, nucleotide metabolism, translation, transcriptional regulation, DNA recombination, protein import into mitochondrial matrix, and nucleosome assembly. Genes annotated with the first three GO terms made up 51% of the 118 genes in the combined module set (Table S4), suggesting the importance of these three biological processes in predicting GBM patient survival. Among them, the role of apoptosis pathway in GBM etiology is much better understood [37] whereas the importance of protein trafficking and degradation pathway is less appreciated. Our result suggest that the latter two pathways also play an important role in GBM patient survival because the discovered modules associated with these two pathways have highly-ranked weights in the SVM classifier (Table S4). A better understanding of these two pathways could ultimately result in personalized therapies for subgroups of GBM patients.

There is increasing evidence that genes controlling protein trafficking play a role in tumor progression and invasion [38]. One of our network modules, module *A*, is highly enriched for genes involved in vesicle trafficking, especially genes of the Rab family of small GTPases (Figure 5A). The Rabs play essential roles in various aspects of membrane traffic control, and like other members of the Ras superfamily, function as molecular switches through changes in its guanine nucleotide binding status. By modulating the trafficking of either growth factor receptors (e.g. EGFR) or cell adhesion molecules (e.g. integrin), Rab proteins can increase the proliferation and invasion potential of tumor cells [39]. Deregulation of Rab expression is associated with multiple cancers [39]. Our data identify that members of four Rab subfamilies, *Rab3*, *Rab8*, *Rab26*, and *Rab27*, are deregulated between LTS- and STS- GBM patients. In addition, they form dense interactions among themselves and other genes involved in protein trafficking. Among those Rabs, members of the brain-enriched Rab, *Rab3*, have the largest presence in module *A*, suggesting that Rab3 genes play a prominent role in GBM patient survival. Thus, they are prime candidates for future detailed studies.

The ordered, temporal degradation of numerous key short-lived regulatory proteins by the proteasome (such as p53, p21, p27, cyclins, cyclin-dependent kinase inhibitors, and tumor suppressors) is required for cell-cycle progression, cell survival, and metastasis. Our result shows modules *H* and *M* are enriched for genes involved in protein degradation through the ubiquitin-proteasome system (Figure 5). Genes in module *H* are enriched for S phase kinase-associated protein 1 (SKP1)-cullin 1 (CUL1)-F-box protein (SCF) family of E3 ubiquitin ligase superfamily [40]. Genes in module *M* are members of the proteasome complex. SCF family ubiquitin ligases promote degradation of diverse substrates, including cell cycle regulatory proteins, transcription factors, and signal transducers. SCF dysfunction has been observed in a number of cancers, including glioblastoma [40].

Because of its fundamental role in protein homeostasis, targeting the ubiquitin-proteasome pathway using proteasome inhibitors represents a novel approach for the treatment of cancer. Clinically, the proteasome inhibitor bortezomib has been used for the treatment of multiple myeloma and mantle cell lymphoma [41]. Clinical trials evaluating the efficacies of proteasome inhibitors for the treatment of solid tumors are in progress [42]. Our discovery that ubiquitin-proteasome pathway is prognostic of GBM patient outcome suggests that targeting this pathway with proteasome inhibitors may be an effective treatment for this deadly disease. Indeed, two proteasome inhibitors, bortezomib [43] and PS-341 [44], have been shown to have anti-proliferation and proapoptotic effects on cell line models of the disease. Additional studies and clinical trials will determine the efficacies of these inhibitors on improving GBM patient outcome.

The relationship between DNA methylation and gene expression is complex. This may help explain the apparent low correlation between them across patient samples. It has been proposed that DNA demethylation is necessary but not sufficient for gene activation. Conversely, methylation of a promoter is not always sufficient for gene repression [31], [45]. In support of this view, Mohn *et al.* found that 21–27% of unmethylated promoters have no detectable expression during mouse neuronal lineage commitment [46]. On the other hand, Fouse *et al.* [45] found that up to 36% of genes in mouse ES cells are still expressed even if methylated in the proximal promoter. Furthermore, 80% of the expressed genes that exhibit promoter methylation are marked by the active histone mark H3K4me3. In this sense, DNA methylation status only provides a precondition for the transcriptional regulatory process and additional factors, such as histone modifications may play more direct and important roles in gene regulation.

In this study, we only considered promoter DNA methylation data. Another major epigenetic regulatory mechanism is covalent modification of histone tail. This mechanism has been shown to operate independently of DNA methylation during tumorigenesis. For instance, H3K27me3 has been found to silence tumor-suppressor genes in cancers that are independently of promoter DNA methylation [47], [48]. Integration of both types of epigenetic data with gene expression and interactome data may lead to improvement on the accuracy of cancer pathway inference algorithms.

## Conclusions

We introduce the MAPIT algorithm to enable principled integration of epigenomic, transcriptomic, and protein interactome data. As a proof-of-principle, we apply MAPIT to discover multi-analyte network markers for the prognosis of glioblastoma multiforme, the most common and aggressive form of brain tumor. MAPIT can be applied to any cancer cohort containing matched data for gene expression and epigenetic profiles. The principle of associating epigenetic data with gene expression and clinical data that differ among samples will be of increasing importance as epigenetic data accumulate. Additionally, the principle of associating cellular state data (i.e. transcriptome and epigenome) with physical interactome can not only help to identify important genes in tumorigenesis, but also provide insight into how they operate. The MAPIT approach is not limited to finding prognostic markers of patient outcome. Indeed, it can be used to identify pathways that relate to any measurable phenotype, such as metastasis and the resistance of tumors to drugs. We anticipate that our approach will make an important contribution toward a basic mechanistic understanding of cancer and in revealing associations of clinical significance.

## Supporting Information

Figure S1 **Survival time of 279 TCGA GBM patients.** Patient clinical data provided by the TCGA consortium. **A**) Boxplot for the survival time distribution of 279 GBM patients. The median survival time is 46.6 weeks. The grey horizontal line indicates the 2 yrs. cutoff used in this study to classify patients into Long Term Survivors (LTS) and Short Term Survivors (STS). **B**) Kaplan-Meier survival curve for LTS and STS GBM patients classified using their patient records.
(DOCX)

Figure S2 **Venn diagram of the three data sets used in this study.** Gene expression and methylation data were obtained from The Cancer Genome Atlas (TCGA) consortium data portal. Protein-protein interaction (PPI) data were compiled from the iRefIndex database and Bandyopadhyay *et al.*
(DOCX)

Figure S3 **Classification accuracies of different module sets.** **A**) GBM patient classification accuracy using network modules generated at different alpha values. Network modules generated at different miPALM alpha values were combined with the SVM-RFE algorithm to identify the final set of features (modules) for classification. Values shown are average of 100 cross validations. eModule, network modules generated using gene expression and PPI interactome. mModule, network modules generated using promoter DNA methylation and PPI interactome. combinedModule, a combination of eModules and mModules that were selected by the SVM-RFE algorithm to be the final set of discriminative features for classification. **B**) Classification accuracy of the combinedModule during feature selection process using the recursive feature elimination algorithm (RFE).
(DOCX)

Figure S4 **Pearson correlation between gene expression and DNA methylation profiles.** Data from 279 GBM patients were used for computing the correlation. All, 8,171 genes in the input network; Diff. expressed: 2,009 differentially expressed genes between LTS and STS GBM patients; Diff. methylated: 1,877 differentially methylated genes; eModule: 156 genes in the set of eModules; Random, 1,877 genes randomly selected from the input network. Values shown in the box are median correlation for each gene set.
(DOCX)

Figure S5 **Cumulative distributions of promoter DNA methylation correlation between random protein pairs and protein pairs that physically interact.** DNA methylation data 279 TCGA patients were used for this analysis. Spearman's rank correlation was calculated for 47,168 pairs of connected proteins in the protein-protein interaction network and the same number of protein pairs randomly picked from the network. P-value is based on one-tailed Kolmogorov-Smirnov test.
(DOCX)

Table S1 **Information of patient data used in this study.** A survival time of two years is used as the cutoff to classify patients into Long Term Survivors (LTS) and Short Term Survivors (STS).
(DOCX)

Table S2 **The set of twenty-five eModules identified using mRNA expression data only.**
(DOCX)

Table S3 **The set of seven mModules identified using DNA methylation data only.**
(DOCX)

Table S4 **The set of multi-analyte modules identified by the MAPIT algorithm.** 38-gene set, set of prognostic genes for GBM patient outcome proposed by Colman *et al.* G-CIMP+ gene set, set of prognostic genes for GBM patient outcome proposed by Noushmehr *et al.* COSMIC genes: genes with somatic mutations in GBM samples documented in the COSMIC database; CNV genes, genes located in Copy Number Variation regions identified by the Cancer Genome Atlas Research Network. Fraction Supported: fraction of module genes overlapping with genes from any of the previous studies. SVM Weight, weights of the final SVM classifier. It indicates the relative importance of each module to the classification.
(DOCX)
