# Supplementary material for: Multi-Analyte Network Markers for Tumor Prognosis
Source: PLoS One. 2012 Dec 26;7(12):e52973. doi: 10.1371/journal.pone.0052973 (PMC3530467; doi:10.1371/journal.pone.0052973)

**Figure S1. Survival time of 279 TCGA GBM patients.** Patient clinical data provided by the TCGA consortium. **A)** Boxplot for the survival time distribution of 279 GBM patients. The median survival time is 46.6 weeks. The grey horizontal line indicates the 2 yrs. cutoff used in this study to classify patients into Long Term Survivors (LTS) and Short Term Survivors (STS). **B**) Kaplan-Meier survival curve for LTS and STS GBM patients classified using their patient records.


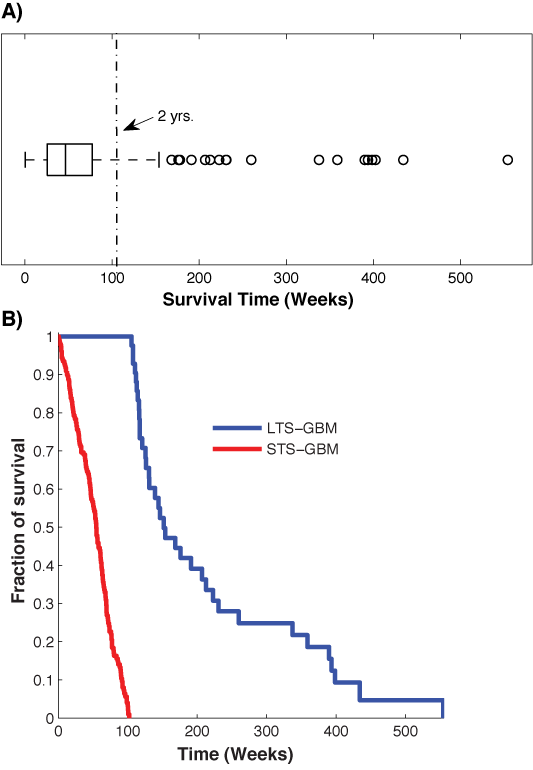

Supplement: Figure S1 — Survival time of 279 TCGA GBM patients. Patient clinical data provided by the TCGA consortium. A) Boxplot for the survival time distribution of 279 GBM patients. The median survival time is 46.6 weeks. The grey horizontal line indicates the 2 yrs. cutoff used in this study to classify patients into Long Term Survivors (LTS) and Short Term Survivors (STS). B) Kaplan-Meier survival curve for LTS and STS GBM patients classified using their patient records. (DOCX) [file pone.0052973.s001.docx]
