# Supplementary material for: Multi-Analyte Network Markers for Tumor Prognosis
Source: PLoS One. 2012 Dec 26;7(12):e52973. doi: 10.1371/journal.pone.0052973 (PMC3530467; doi:10.1371/journal.pone.0052973)

**Figure S2.** Venn diagram of the three data sets used in this study. Gene expression and methylation data were obtained from The Cancer Genome Atlas (TCGA) consortium data portal. Protein-protein interaction (PPI) data were compiled from the iRefIndex database [[1](#_ENREF_1)] and Bandyopadhyay *et al.* [[2](#_ENREF_2)]*.*


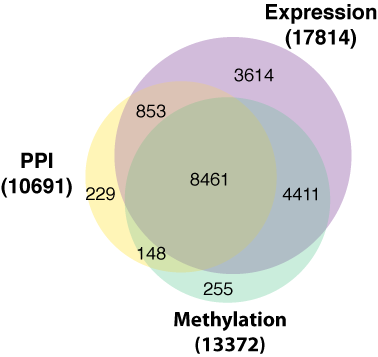

Supplement: Figure S2 — Venn diagram of the three data sets used in this study. Gene expression and methylation data were obtained from The Cancer Genome Atlas (TCGA) consortium data portal. Protein-protein interaction (PPI) data were compiled from the iRefIndex database and Bandyopadhyay et al. (DOCX) [file pone.0052973.s002.docx]
