# Supplementary material for: Multi-Analyte Network Markers for Tumor Prognosis
Source: PLoS One. 2012 Dec 26;7(12):e52973. doi: 10.1371/journal.pone.0052973 (PMC3530467; doi:10.1371/journal.pone.0052973)

**Figure S3. A) GBM patient classification accuracy using network modules generated at different alpha values.** Network modules generated at different miPALM alpha values were combined with the SVM-RFE algorithm to identify the final set of features (modules) for classification. Values shown are average of 100 cross validations. eModule, network modules generated using gene expression and PPI interactome. mModule, network modules generated using promoter DNA methylation and PPI interactome. combinedModule, a combination of eModules and mModules that were selected by the SVM-RFE algorithm to be the final set of discriminative features for classification. **B)** **Classification accuracy of the combinedModule during feature selection process using the recursive feature elimination algorithm (RFE).**


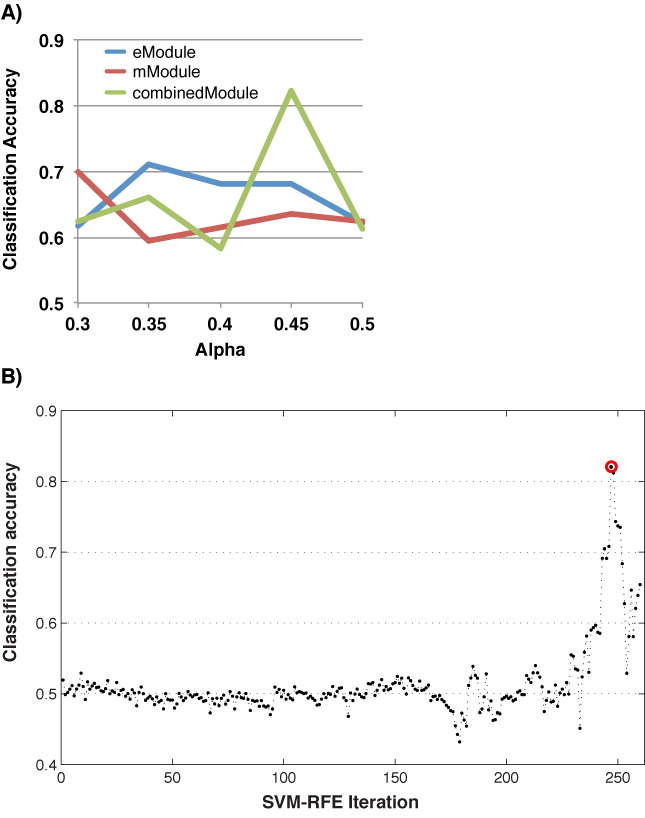

Supplement: Figure S3 — Classification accuracies of different module sets. A) GBM patient classification accuracy using network modules generated at different alpha values. Network modules generated at different miPALM alpha values were combined with the SVM-RFE algorithm to identify the final set of features (modules) for classification. Values shown are average of 100 cross validations. eModule, network modules generated using gene expression and PPI interactome. mModule, network modules generated using promoter DNA methylation and PPI interactome. combinedModule, a combination of eModules and mModules that were selected by the SVM-RFE algorithm to be the final set of discriminative features for classification. B) Classification accuracy of the combinedModule during feature selection process using the recursive feature elimination algorithm (RFE). (DOCX) [file pone.0052973.s003.docx]
