# Supplementary material for: Multi-Analyte Network Markers for Tumor Prognosis
Source: PLoS One. 2012 Dec 26;7(12):e52973. doi: 10.1371/journal.pone.0052973 (PMC3530467; doi:10.1371/journal.pone.0052973)

**Figure S4. Pearson correlation between gene expression and DNA methylation profiles.** Data from 279 GBM patients were used for computing the correlation. All, 8,171 genes in the input network; Diff. expressed: 2,009 differentially expressed genes between LTS and STS GBM patients; Diff. methylated: 1,877 differentially methylated genes; eModule: 156 genes in the set of eModules; Random, 1,877 genes randomly selected from the input network. Values shown in the box are median correlation for each gene set.


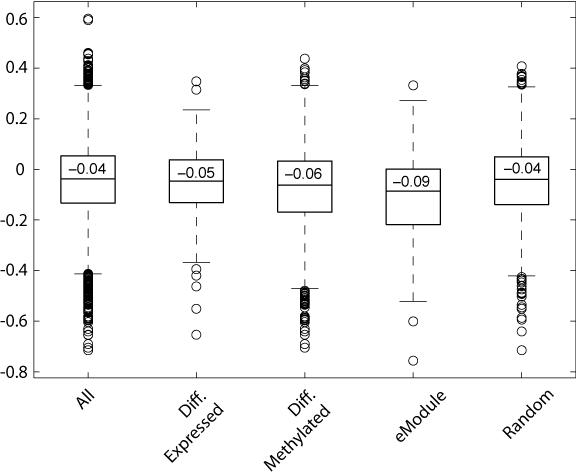

Supplement: Figure S4 — Pearson correlation between gene expression and DNA methylation profiles. Data from 279 GBM patients were used for computing the correlation. All, 8,171 genes in the input network; Diff. expressed: 2,009 differentially expressed genes between LTS and STS GBM patients; Diff. methylated: 1,877 differentially methylated genes; eModule: 156 genes in the set of eModules; Random, 1,877 genes randomly selected from the input network. Values shown in the box are median correlation for each gene set. (DOCX) [file pone.0052973.s004.docx]
