# Supplementary material for: Multi-Analyte Network Markers for Tumor Prognosis
Source: PLoS One. 2012 Dec 26;7(12):e52973. doi: 10.1371/journal.pone.0052973 (PMC3530467; doi:10.1371/journal.pone.0052973)
Supplement: Figure S5 — Cumulative distributions of promoter DNA methylation correlation between random protein pairs and protein pairs that physically interact. DNA methylation data 279 TCGA patients were used for this analysis. Spearman's rank correlation was calculated for 47,168 pairs of connected proteins in the protein-protein interaction network and the same number of protein pairs randomly picked from the network. P-value is based on one-tailed Kolmogorov-Smirnov test. (DOCX) [file pone.0052973.s005.docx]

**Figure S5.** **Cumulative distributions of promoter DNA methylation correlation between random protein pairs and protein pairs that physically interact.** DNA methylation data 279 TCGA patients were used for this analysis. Spearman’s rank correlation was calculated for 47,168 pairs of connected proteins in the protein-protein interaction network and the same number of protein pairs randomly picked from the network. P-value is based on one-tailed Kolmogorov-Smirnov test.

**
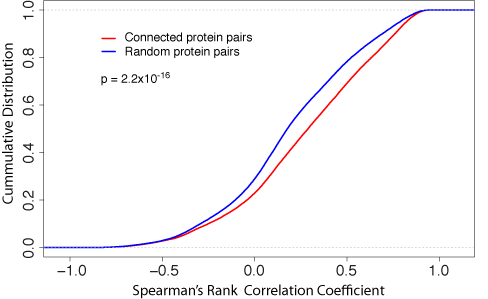
**
