# Supplementary material for: Multi-Analyte Network Markers for Tumor Prognosis
Source: PLoS One. 2012 Dec 26;7(12):e52973. doi: 10.1371/journal.pone.0052973 (PMC3530467; doi:10.1371/journal.pone.0052973)
Supplement: Table S1 — Information of patient data used in this study. A survival time of two years is used as the cutoff to classify patients into Long Term Survivors (LTS) and Short Term Survivors (STS). (DOCX) [file pone.0052973.s006.docx]

**Table S1. Information of patient data used in this study.** A survival time of two years is used as the cutoff to classify patients into Long Term Survivors (LTS) and Short Term Survivors (STS).

| Patient barcode | Classification label  (LTS=1, STS=2) | Survival time (days) |
| --- | --- | --- |
| TCGA-12-1088 | 1 | 3880 |
| TCGA-02-0114 | 1 | 3040 |
| TCGA-02-0113 | 1 | 2817 |
| TCGA-12-0818 | 1 | 2790 |
| TCGA-02-0028 | 1 | 2755 |
| TCGA-02-0080 | 1 | 2729 |
| TCGA-02-0014 | 1 | 2511 |
| TCGA-02-0021 | 1 | 2361 |
| TCGA-12-3644 | 1 | 1818 |
| TCGA-15-1447 | 1 | 1618 |
| TCGA-02-0024 | 1 | 1614 |
| TCGA-02-0085 | 1 | 1560 |
| TCGA-02-0116 | 1 | 1489 |
| TCGA-06-0125 | 1 | 1448 |
| TCGA-12-3646 | 1 | 1338 |
| TCGA-14-1456 | 1 | 1245 |
| TCGA-27-1834 | 1 | 1232 |
| TCGA-12-0827 | 1 | 1179 |
| TCGA-02-0010 | 1 | 1077 |
| TCGA-12-3652 | 1 | 1062 |
| TCGA-06-0129 | 1 | 1024 |
| TCGA-12-1091 | 1 | 1010 |
| TCGA-14-1402 | 1 | 974 |
| TCGA-14-1454 | 1 | 917 |
| TCGA-27-1836 | 1 | 914 |
| TCGA-16-1045 | 1 | 882 |
| TCGA-14-0871 | 1 | 879 |
| TCGA-02-0069 | 1 | 873 |
| TCGA-12-0826 | 1 | 845 |
| TCGA-02-0102 | 1 | 821 |
| TCGA-12-3648 | 1 | 819 |
| TCGA-32-2498 | 1 | 818 |
| TCGA-06-1800 | 1 | 815 |
| TCGA-06-0137 | 1 | 812 |
| TCGA-14-0866 | 1 | 801 |
| TCGA-16-0849 | 1 | 793 |
| TCGA-12-0670 | 1 | 790 |
| TCGA-12-1599 | 1 | 781 |
| TCGA-19-1390 | 1 | 772 |
| TCGA-12-0819 | 1 | 753 |
| TCGA-27-2518 | 1 | 752 |
| TCGA-27-1833 | 1 | 737 |
| TCGA-12-0822 | 2 | 715 |
| TCGA-02-0007 | 2 | 705 |
| TCGA-14-1451 | 2 | 703 |
| TCGA-14-0786 | 2 | 701 |
| TCGA-06-0879 | 2 | 699 |
| TCGA-02-0083 | 2 | 691 |
| TCGA-06-0128 | 2 | 691 |
| TCGA-12-1597 | 2 | 675 |
| TCGA-19-0957 | 2 | 665 |
| TCGA-12-1092 | 2 | 661 |
| TCGA-27-1835 | 2 | 648 |
| TCGA-16-1062 | 2 | 646 |
| TCGA-32-1973 | 2 | 641 |
| TCGA-06-0939 | 2 | 637 |
| TCGA-02-0075 | 2 | 634 |
| TCGA-32-2494 | 2 | 632 |
| TCGA-02-0011 | 2 | 630 |
| TCGA-12-0829 | 2 | 626 |
| TCGA-06-0124 | 2 | 619 |
| TCGA-02-0057 | 2 | 604 |
| TCGA-02-0064 | 2 | 600 |
| TCGA-14-1037 | 2 | 587 |
| TCGA-15-1446 | 2 | 581 |
| TCGA-12-0820 | 2 | 562 |
| TCGA-02-0006 | 2 | 558 |
| TCGA-02-0043 | 2 | 556 |
| TCGA-14-1823 | 2 | 542 |
| TCGA-06-0147 | 2 | 541 |
| TCGA-14-1821 | 2 | 540 |
| TCGA-02-0107 | 2 | 537 |
| TCGA-16-0848 | 2 | 535 |
| TCGA-14-2554 | 2 | 532 |
| TCGA-02-0089 | 2 | 515 |
| TCGA-32-1991 | 2 | 515 |
| TCGA-02-2466 | 2 | 511 |
| TCGA-27-1831 | 2 | 504 |
| TCGA-14-0865 | 2 | 502 |
| TCGA-19-2629 | 2 | 501 |
| TCGA-16-0850 | 2 | 498 |
| TCGA-02-2486 | 2 | 492 |
| TCGA-27-2523 | 2 | 489 |
| TCGA-12-1093 | 2 | 486 |
| TCGA-14-1034 | 2 | 485 |
| TCGA-32-2615 | 2 | 485 |
| TCGA-12-1095 | 2 | 482 |
| TCGA-32-1978 | 2 | 482 |
| TCGA-27-2528 | 2 | 479 |
| TCGA-02-0115 | 2 | 476 |
| TCGA-12-1598 | 2 | 476 |
| TCGA-02-2485 | 2 | 469 |
| TCGA-32-1970 | 2 | 467 |
| TCGA-02-2483 | 2 | 465 |
| TCGA-12-3649 | 2 | 463 |
| TCGA-14-0736 | 2 | 459 |
| TCGA-32-2495 | 2 | 456 |
| TCGA-32-1987 | 2 | 451 |
| TCGA-12-1600 | 2 | 448 |
| TCGA-02-0047 | 2 | 447 |
| TCGA-12-1097 | 2 | 442 |
| TCGA-12-3653 | 2 | 442 |
| TCGA-27-2527 | 2 | 438 |
| TCGA-06-0133 | 2 | 435 |
| TCGA-19-0963 | 2 | 434 |
| TCGA-02-0034 | 2 | 430 |
| TCGA-27-1837 | 2 | 427 |
| TCGA-16-1056 | 2 | 426 |
| TCGA-16-1063 | 2 | 425 |
| TCGA-26-1439 | 2 | 422 |
| TCGA-14-0790 | 2 | 418 |
| TCGA-41-2572 | 2 | 406 |
| TCGA-15-1449 | 2 | 404 |
| TCGA-32-4208 | 2 | 400 |
| TCGA-06-0130 | 2 | 394 |
| TCGA-19-1388 | 2 | 394 |
| TCGA-02-2470 | 2 | 393 |
| TCGA-12-3651 | 2 | 386 |
| TCGA-32-1986 | 2 | 386 |
| TCGA-19-1787 | 2 | 385 |
| TCGA-02-0052 | 2 | 383 |
| TCGA-32-4211 | 2 | 383 |
| TCGA-06-2558 | 2 | 380 |
| TCGA-14-1459 | 2 | 378 |
| TCGA-12-1094 | 2 | 372 |
| TCGA-32-2491 | 2 | 372 |
| TCGA-02-0027 | 2 | 370 |
| TCGA-06-0139 | 2 | 362 |
| TCGA-19-0955 | 2 | 358 |
| TCGA-06-0143 | 2 | 357 |
| TCGA-32-4213 | 2 | 356 |
| TCGA-02-0001 | 2 | 353 |
| TCGA-27-1838 | 2 | 350 |
| TCGA-28-1745 | 2 | 344 |
| TCGA-14-0789 | 2 | 342 |
| TCGA-32-4209 | 2 | 338 |
| TCGA-12-3650 | 2 | 333 |
| TCGA-32-4719 | 2 | 330 |
| TCGA-19-1385 | 2 | 327 |
| TCGA-02-0038 | 2 | 326 |
| TCGA-12-0821 | 2 | 323 |
| TCGA-02-0009 | 2 | 322 |
| TCGA-06-0155 | 2 | 318 |
| TCGA-27-2519 | 2 | 316 |
| TCGA-27-2521 | 2 | 316 |
| TCGA-06-0141 | 2 | 313 |
| TCGA-16-1055 | 2 | 313 |
| TCGA-02-0074 | 2 | 310 |
| TCGA-06-0148 | 2 | 307 |
| TCGA-26-1438 | 2 | 305 |
| TCGA-27-1832 | 2 | 300 |
| TCGA-26-1440 | 2 | 296 |
| TCGA-19-2619 | 2 | 294 |
| TCGA-41-2575 | 2 | 290 |
| TCGA-06-2570 | 2 | 285 |
| TCGA-26-1799 | 2 | 285 |
| TCGA-06-2561 | 2 | 282 |
| TCGA-06-0875 | 2 | 279 |
| TCGA-28-1749 | 2 | 279 |
| TCGA-16-1060 | 2 | 278 |
| TCGA-12-1096 | 2 | 277 |
| TCGA-19-1386 | 2 | 276 |
| TCGA-41-2573 | 2 | 272 |
| TCGA-06-0876 | 2 | 271 |
| TCGA-12-0828 | 2 | 271 |
| TCGA-32-2634 | 2 | 270 |
| TCGA-32-2632 | 2 | 269 |
| TCGA-02-0086 | 2 | 268 |
| TCGA-06-1801 | 2 | 263 |
| TCGA-19-0960 | 2 | 262 |
| TCGA-06-2563 | 2 | 259 |
| TCGA-28-1752 | 2 | 257 |
| TCGA-02-0058 | 2 | 254 |
| TCGA-28-1750 | 2 | 253 |
| TCGA-14-2555 | 2 | 242 |
| TCGA-06-1802 | 2 | 239 |
| TCGA-14-1825 | 2 | 232 |
| TCGA-27-2524 | 2 | 231 |
| TCGA-28-1751 | 2 | 231 |
| TCGA-12-1090 | 2 | 230 |
| TCGA-19-2623 | 2 | 229 |
| TCGA-06-1084 | 2 | 226 |
| TCGA-32-2638 | 2 | 224 |
| TCGA-32-2616 | 2 | 223 |
| TCGA-28-2513 | 2 | 222 |
| TCGA-06-0878 | 2 | 218 |
| TCGA-14-1829 | 2 | 217 |
| TCGA-19-1786 | 2 | 217 |
| TCGA-14-1452 | 2 | 216 |
| TCGA-26-1443 | 2 | 216 |
| TCGA-19-2631 | 2 | 213 |
| TCGA-06-0126 | 2 | 210 |
| TCGA-02-0046 | 2 | 208 |
| TCGA-06-1086 | 2 | 208 |
| TCGA-06-2565 | 2 | 207 |
| TCGA-12-1602 | 2 | 205 |
| TCGA-28-2506 | 2 | 205 |
| TCGA-06-0877 | 2 | 204 |
| TCGA-14-1458 | 2 | 202 |
| TCGA-02-0054 | 2 | 199 |
| TCGA-16-1460 | 2 | 195 |
| TCGA-14-0783 | 2 | 188 |
| TCGA-06-0122 | 2 | 187 |
| TCGA-02-0060 | 2 | 183 |
| TCGA-41-3915 | 2 | 183 |
| TCGA-06-2566 | 2 | 182 |
| TCGA-06-2564 | 2 | 181 |
| TCGA-19-1387 | 2 | 181 |
| TCGA-14-1827 | 2 | 179 |
| TCGA-06-0881 | 2 | 178 |
| TCGA-12-1089 | 2 | 177 |
| TCGA-02-0071 | 2 | 167 |
| TCGA-06-0882 | 2 | 165 |
| TCGA-14-0817 | 2 | 164 |
| TCGA-28-2514 | 2 | 160 |
| TCGA-27-1830 | 2 | 154 |
| TCGA-19-1790 | 2 | 153 |
| TCGA-06-2559 | 2 | 149 |
| TCGA-19-2620 | 2 | 148 |
| TCGA-28-2509 | 2 | 145 |
| TCGA-02-0003 | 2 | 144 |
| TCGA-28-1760 | 2 | 143 |
| TCGA-32-1982 | 2 | 142 |
| TCGA-19-1389 | 2 | 141 |
| TCGA-16-1047 | 2 | 139 |
| TCGA-19-4068 | 2 | 136 |
| TCGA-41-3393 | 2 | 135 |
| TCGA-06-2567 | 2 | 133 |
| TCGA-16-0861 | 2 | 130 |
| TCGA-06-1805 | 2 | 127 |
| TCGA-12-1099 | 2 | 125 |
| TCGA-19-2625 | 2 | 124 |
| TCGA-06-1087 | 2 | 123 |
| TCGA-12-1098 | 2 | 121 |
| TCGA-16-0846 | 2 | 119 |
| TCGA-14-1401 | 2 | 114 |
| TCGA-14-3477 | 2 | 114 |
| TCGA-32-4210 | 2 | 112 |
| TCGA-19-1392 | 2 | 111 |
| TCGA-19-1788 | 2 | 111 |
| TCGA-02-0037 | 2 | 109 |
| TCGA-02-0099 | 2 | 106 |
| TCGA-14-4157 | 2 | 103 |
| TCGA-19-0964 | 2 | 103 |
| TCGA-06-0169 | 2 | 100 |
| TCGA-14-0812 | 2 | 99 |
| TCGA-19-1789 | 2 | 93 |
| TCGA-27-2526 | 2 | 87 |
| TCGA-02-0033 | 2 | 86 |
| TCGA-06-2562 | 2 | 86 |
| TCGA-28-1756 | 2 | 86 |
| TCGA-28-1747 | 2 | 77 |
| TCGA-02-0055 | 2 | 76 |
| TCGA-06-0145 | 2 | 71 |
| TCGA-14-0787 | 2 | 68 |
| TCGA-14-0867 | 2 | 62 |
| TCGA-14-1795 | 2 | 59 |
| TCGA-28-1755 | 2 | 46 |
| TCGA-14-0813 | 2 | 41 |
| TCGA-28-1753 | 2 | 36 |
| TCGA-14-1453 | 2 | 35 |
| TCGA-14-1396 | 2 | 34 |
| TCGA-06-2557 | 2 | 33 |
| TCGA-19-2621 | 2 | 33 |
| TCGA-14-1794 | 2 | 30 |
| TCGA-41-3392 | 2 | 30 |
| TCGA-14-1455 | 2 | 28 |
| TCGA-41-2571 | 2 | 26 |
| TCGA-19-0962 | 2 | 20 |
| TCGA-28-2502 | 2 | 20 |
| TCGA-32-1976 | 2 | 15 |
| TCGA-06-2569 | 2 | 13 |
| TCGA-14-3476 | 2 | 12 |
| TCGA-28-1746 | 2 | 6 |
| TCGA-41-4097 | 2 | 6 |
| TCGA-19-2624 | 2 | 5 |
| TCGA-19-1791 | 2 | 4 |
| TCGA-28-1757 | 2 | 4 |
| TCGA-32-1977 | 2 | 7 |
