# Supplementary material for: Multi-Analyte Network Markers for Tumor Prognosis
Source: PLoS One. 2012 Dec 26;7(12):e52973. doi: 10.1371/journal.pone.0052973 (PMC3530467; doi:10.1371/journal.pone.0052973)
Supplement: Table S2 — The set of twenty-five eModules identified using mRNA expression data only. (DOCX) [file pone.0052973.s007.docx]

**Table S2. The set of twenty-five eModules identified using mRNA expression data only.**

| Module ID | Module genes | GO pvalue | GO Term Description |
| --- | --- | --- | --- |
| 1 | *BCL7A SFPQ HDAC6 EPRS NFKBIE NONO RELB BCL3* | 9.79E-04 | follicular dendritic cell differentiation |
| 2 | *RPL23A RPL26 RPL32 OPN4 TMEM87A PAK2* | 1.09E-05 | viral reproduction |
| 3 | *SAT1 CRMP1 FAM86C INO80C TMEM141 TCF25 HSPE1 B4GALT3* | 3.91E-03 | polyamine biosynthetic process |
| 4 | *SECTM1 SPN CD7 C1GALT1C1 CUBN PI4K2A SUSD2 GIF LGALS1 LGALS3 LGALS3BP* | 4.94E-06 | cobalamin transport |
| 5 | *SFPQ SNRPA C19orf43 PTBP1* | 3.25E-05 | RNA splicing |
| 6 | *SFRS1 SNRNP70 SRPK1 SRPK2 DDHD1 CLK1 LUC7L3 PRM1 PSIP1* | 1.91E-05 | RNA splicing |
| 7 | *POLR3D POLR3K POLR1C POLR1D POLR3A POLR3E POLR3B* | 6.78E-12 | transcription from RNA polymerase III promoter |
| 8 | *SNCA ELK1 LAMP2 MAPK8* | 1.46E-04 | stress-activated MAPK cascade |
| 9 | *STAT3 DAXX LSP1 PML* | 1.16E-04 | interspecies interaction between organisms |
| 10 | *UBQLN4 UBQLN1 CSTF2T SMCR7* | 1.96E-03 | regulation of protein ubiquitination |
| 11 | *SPP1 UBQLN4 UBQLN2 HERPUD1 PLUNC PDLIM7 CEND1 ARL4C PPIC QSOX1* | 8.31E-04 | ossification |
| 12 | *WAS SNX9 SNX33 WIPF2 AMD1* | 2.55E-04 | cell communication |
| 13 | *WHSC1L1 CBX1 CBX3 CBX5 HIST3H2A HIST3H2BB NSL1 PRR14 ARL5A MKI67* | 1.54E-03 | nucleosome assembly |
| 14 | *CASP10 CASP8 CFLAR DEDD2 DEDD* | 5.67E-07 | regulation of apoptosis |
| 15 | *VAV3 SH3BGRL3 DAPP1 TNS3 CRKL SH2B3 EGF ERBB2 ERBB3 PIK3R2* | 4.04E-06 | negative regulation of secretion |
| 16 | *MAPKSP1 HNF4G MAP2K1 MAPK1* | 9.44E-05 | activation of MAPKK activity |
| 17 | *MBD3L1 GATAD2A GATAD2B MBD2 MBD3 MTA2* | 6.07E-04 | negative regulation of transcription from RNA polymerase II promoter |
| 18 | *CDC25A DNAJC27 MAP3K5 MAP3K6 RAF1* | 6.26E-05 | activation of JUN kinase activity |
| 19 | *DBF4 CDC7 DBF4B CHAF1B ORC5L* | 1.97E-05 | DNA replication |
| 20 | *CDC5L RBM16 MCM4 ORC1L* | 6.35E-03 | DNA-dependent DNA replication initiation |
| 21 | *DCLRE1C C1D ILF2 ILF3 PRKDC* | 2.55E-04 | telomere maintenance |
| 22 | *TNIP3 NFKBIZ IRF8 IRF1 NFKB1* | 1.38E-04 | cellular response to mechanical stimulus |
| 23 | *SAFB GPRC5C BAT5 MRPL37 FRMD4A HNRNPA1 ILF2 ILF3 NNT ATP5G3 PLOD2* | 1.35E-03 | nuclear import |
| 24 | *ZC3H11A MLH1 MSH2 MSH6 PMS1* | 3.67E-11 | mismatch repair |
| 25 | *IL13 IL13RA1 IL13RA2 IL4 IL4R* | 3.00E-07 | regulation of proton transport |
