# Supplementary material for: Multi-Analyte Network Markers for Tumor Prognosis
Source: PLoS One. 2012 Dec 26;7(12):e52973. doi: 10.1371/journal.pone.0052973 (PMC3530467; doi:10.1371/journal.pone.0052973)
Supplement: Table S3 — The set of seven mModules identified using DNA methylation data only. (DOCX) [file pone.0052973.s008.docx]

**Table S3. The set of seven mModules identified using DNA methylation data only.**

| Module ID | Module genes | GO pvalue | GO Term Description |
| --- | --- | --- | --- |
| 1 | SPTAN1 GCNT1 LMNB1 PRKCB | 1.69E-04 | cellular component disassembly involved in apoptosis |
| 2 | SSBP1 TAL1 HDAC8 RUNX1T1 CBFA2T2 CBFA2T3 SSBP3 ZNF652 | 5.07E-04 | regulation of transcription, DNA-dependent |
| 3 | CASP10 CASP8 CFLAR DEDD2 DEDD | 5.67E-07 | regulation of apoptosis |
| 4 | CAV1 CAV2 GJB2 AQP3 PLD2 | 3.00E-07 | caveola assembly |
| 5 | POLR3F CALCRL CRCP POLR2H | 1.44E-07 | transcription from RNA polymerase III promoter |
| 6 | MED12 MED8 MED13L MED26 MED9 MED19 | 1.92E-08 | regulation of transcription from RNA polymerase II promoter |
| 7 | BID BAK1 BAX BCL2A1 BCL2L10 BCL2L2 | 1.49E-07 | release of cytochrome c from mitochondria |
