# Supplementary material for: Multi-Analyte Network Markers for Tumor Prognosis
Source: PLoS One. 2012 Dec 26;7(12):e52973. doi: 10.1371/journal.pone.0052973 (PMC3530467; doi:10.1371/journal.pone.0052973)
Supplement: Table S4 — The set of multi-analyte modules identified by the MAPIT algorithm. 38-gene set, set of prognostic genes for GBM patient outcome proposed by Colman et al. G-CIMP+ gene set, set of prognostic genes for GBM patient outcome proposed by Noushmehr et al. COSMIC genes: genes with somatic mutations in GBM samples documented in the COSMIC database; CNV genes, genes located in Copy Number Variation regions identified by the Cancer Genome Atlas Research Network. Fraction Supported: fraction of module genes overlapping with genes from any of the previous studies. SVM Weight, weights of the final SVM classifier. It indicates the relative importance of each module to the classification. (DOCX) [file pone.0052973.s009.docx]

**Table S4. The set of multi-analyte modules identified by the MAPIT algorithm.** 38-gene set, set of prognostic genes for GBM patient outcome proposed by Colman *et al*. G-CIMP+ gene set, set of prognostic genes for GBM patient outcome proposed by Noushmehr *et al*. COSMIC genes: genes with somatic mutations in GBM samples documented in the COSMIC database [[3](#_ENREF_3)]; CNV genes, genes located in Copy Number Variation regions identified by the Cancer Genome Atlas Research Network [[4](#_ENREF_4)]. Fraction Supported: fraction of module genes overlapping with genes from any of the previous studies. SVM Weight, weights of the final SVM classifier. It indicates the relative importance of each module to the classification.

| Module ID | Module genes | 38-gene set genes | G-CIMP+ genes | COSMIC genes | CNV-genes | Fraction Supported | GO pvalue | GO Term Description | SVM Weight |
| --- | --- | --- | --- | --- | --- | --- | --- | --- | --- |
| A | *RPH3AL BSN WDR7 CACNA1S RAB26 SYTL4 RAB3IP RPH3A RAB3GAP1 RIMS2 UNC13D ADD2 DMXL2 MLPH RAB3C EXPH5 RAB8A PIGR RAB27A RAB27B RAB3A RAB3B RAB3D* | *none* | *RAB27B RAB3D* | *BSN CACNA1S RPH3A RIMS2 RAB3C* | *RAB3D* | 0.30 | 5.10E-14 | Protein trafficking | 3.66 |
| B | *RPL10 RPL10A RPL19 RPL23A RPL35 MRPL3 RPS14 MRPL15 OPN4 AK2* | *none* | *none* | *RPS14* | *none* | 0.10 | 2.15E-12 | Translation | 3.13 |
| C | *SEL1L SON TNFRSF1A TRADD DNAJC6 CARD10 CLTC CLTCL1 C9orf89 FXYD7 ATP1A1 BCL10* | *none* | *none* | *none* | *SEL1L CLTCL1* | 0.17 | 2.66E-04 | TNF signaling pathway/Apoptosis | 3.70 |
| D | *SIAH2 SYP PEG10 C10orf10 C3orf15 AKAP14 AKAP1 MYCBP PEG3 PRKAR2B* | *none* | *C10orf10 AKAP1* | *SYP PEG10* | *PEG3* | 0.50 | 1.22E-03 | Apoptosis | 3.75 |
| E | *TPT1 ARFGEF1 UBE2J2 GRPEL1 ATP5F1 RAD54L* | *none* | *none* | *none* | *none* | 0.00 | 7.34E-04 | Protein import into mitochondrial matrix | 1.52 |
| F | *VPS72 UMPS ZMPSTE24 ATP2C1 CYB5R1 CAD ADSSL1 CYB5R2 CARKD FAM58A OMA1 GART GGPS1 HAGH ARG1* | *none* | *CYB5R1 ADSSL1 CYB5R2* | *ADSSL1 GGPS1* | *CARKD* | 0.33 | 1.48E-06 | Nucleotide metabolic process | 2.47 |
| G | *MYST3 HDAC8 RUNX1T1 CBFA2T2 CBFA2T3 TRIM22 ZNF652 ECM1 RERE* | *none* | *none* | *MYST3 RUNX1T1 RERE* | *RERE* | 0.33 | 7.78E-05 | Transcription regulation | 2.65 |
| H | *FBXO4 FBXO7 FBXL3 FBXO18 SUGT1 CDC34 CUL1 NEDD8 RBX1* | *none* | *none* | *none* | *CDC34* | 0.11 | 1.42E-10 | Ubiquitin-dependent protein catabolic process | 4.60 |
| I | *HIST1H2BK NOP56 NOL6 HIST1H2BD HIRA* | *none* | *none* | *none* | *HIRA* | 0.20 | 3.48E-04 | Nucleosome assembly | 2.39 |
| J | *HIST2H2AB KBTBD7 WDR76 MSH2 MSH3 MSH6 NBN BARD1 RAD50* | *none* | *none* | *MSH2 MSH6 NBN* | *none* | 0.33 | 3.69E-09 | DNA recombination | 3.63 |
| K | *RPL34 RPL35 RPL36AL RPL26L1 CDC42BPB* | *none* | *none* | *none* | *none* | 0.00 | 1.15E-06 | Translation | 2.68 |
| L | *MYST3 HDAC8 RUNX1T1 CBFA2T2 CBFA2T3 TRIM22 ZNF652 ECM1 RERE* | *none* | *none* | *MYST3 RUNX1T1 RERE* | *RERE* | 0.33 | 7.78E-05 | Regulation of transcription | 2.05 |
| M | *PSMD14 MFSD10 RBM15B KPNB1 PSMC2 PSMC4* | *none* | *MFSD10* | *none* | *none* | 0.17 | 2.27E-06 | Protein catabolic process | 4.34 |
